# Emotion crafting links parental autonomy support and warmth to young adult well-being

**DOI:** 10.3389/fpsyg.2025.1629350

**Published:** 2025-10-15

**Authors:** Nureda Taşkesen, Maria Elena Hernandez Hernandez, Bertus F. Jeronimus, Jolene Van der Kaap-Deeder

**Affiliations:** ^1^Department of Psychology, Norwegian University of Science and Technology, Trondheim, Norway; ^2^Department of Developmental Psychology, University of Groningen, Groningen, Netherlands; ^3^Department of Clinical Psychology, Utrecht University, Utrecht, Netherlands

**Keywords:** proactive emotion regulation, positive emotions, psychological well-being, self-determination theory, broaden-and-build theory

## Abstract

Emotion crafting, defined as individuals’ awareness of positive emotion-inducing situations and their proactive efforts to seek them out, partly explains how parental autonomy support and warmth relate to young adults’ well-being. Data from 254 young adults (69.3% women; *M*_age_ = 22.70; *SD*_age_ = 2.07) in Norway and Germany showed that perceived parental autonomy support and warmth related to well-being (i.e., resilience and flourishing), and that these relations were mediated by emotion crafting (i.e., awareness and action). However, emotion crafting action and well-being were no longer linked after controlling for savoring beliefs. Stronger associations between parental warmth and emotion crafting awareness and between emotion crafting action and flourishing were observed in Norwegian (compared to German) young adults. Maternal parenting was more strongly associated with well-being than paternal parenting. Overall, these findings highlight the role of emotion crafting in linking parenting practices to young adults’ well-being.

## 1 Introduction

### 1.1 Parenting and well-being in youth

Parenting practices include strategies to nurture and guide children and have long-lasting effects on the offspring’s health and well-being, academic success, relationships, and social networks (Morris et al., 2021; Van der Kaap-Deeder et al., 2017; Yu et al., 2019). Among all the parenting strategies, parental autonomy support and warmth are consistently associated with positive developmental outcomes. Autonomy-supportive parents validate the child’s perspective and encourage authentic decision-making, whereas warm parents foster affectionate and attuned interactions such as through hugs, kisses, or holding one close, and engage in positive, kind emotional exchanges (Soenens et al., 2017).

Autonomy-supportive parenting is associated with higher adolescent self-esteem and fewer depressive symptoms during school transitions (middle, high, and post-high school; see Duineveld et al., 2017). Both adolescents and young adults who report more parental autonomy support also report less loneliness and depressive symptoms (Barber et al., 1994; Inguglia et al., 2015) and more vitality and fulfillment of their autonomy, competence, and relatedness needs (Costa et al., 2016).

Warm parenting fosters children’s psychological adjustment and prosocial behaviors, such as generosity and empathy, and is associated with adult health and well-being (see meta-analysis by Khaleque, 2013) and stress resilience such as during academic pressure (Quach et al., 2015). Moreover, parental warmth predicts flourishing of their child into mid-life and reduced risk behavior such as drug use and smoking (Chen et al., 2019).

Parenting is often examined in childhood and adolescence, but growing evidence suggests that parenting practices continue to shape individuals along the adult lifespan (Nelson, 2022). In many Western European countries, the transition to adulthood has become increasingly delayed, complex, and extended by prolonged education, postponed family formation, and continued ties to the parental home (Billari and Liefbroer, 2010). This prolonged transition implies that young adults may remain emotionally and financially dependent on their parents and continue to be influenced by both earlier and ongoing parenting practices. Supporting this view, Green et al. (2024) found direct associations between positive parenting and young adults’ psychological need satisfaction, as well as indirect links to their well-being. Accordingly, it remains important to investigate how perceived parental autonomy support and warmth relate to young adults’ well-being and emotion regulation.

### 1.2 Parenting and emotion regulation in youth

One pathway through which these parenting practices contribute to well-being is by influencing the development of adaptive emotion regulation strategies in offspring (Brenning et al., 2015; Bülow et al., 2022; Jaffe et al., 2010; Morris et al., 2017). Emotion regulation refers to how individuals shape the emotions they experience and when and why they feel and express these emotions (Gross, 2015).

Autonomy-supportive parenting can foster an environment where children are encouraged to explore and express their emotions at their own pace, while offering guidance and respecting their emotional needs (Brenning et al., 2015). By explaining the rationale behind their actions, validating the child’s feelings, and avoiding controlling language (e.g., “must” or “should”), autonomy-supportive parents help children develop a stronger sense of self and emotional understanding (Slemp et al., 2024). Similarly, parental warmth can promote a secure and emotionally supportive environment where parents display effective emotional expressions, promote secure attachment, and guide their children to manage their emotions (Yavuz et al., 2022).

Empirical research support these claims, showing that both parental autonomy support and warmth are linked to the development of adaptive emotion regulation skills (Bai et al., 2016; Butterfield et al., 2021; Ryan et al., 2016). Children are thought to observe parental emotion regulation and to be influenced by parental acceptance of and reaction to emotions and coaching of the child (Morris et al., 2007, 2017). Adolescents who perceived their mothers as autonomy-supportive reported lower emotion suppression and higher integrative emotion regulation 1 year later (Brenning et al., 2015). This form of regulation, characterized by openness towards and acceptance of emotions, was in turn associated with higher self-esteem and to fewer depressive symptoms (Brenning et al., 2015). Parental autonomy support was also linked to greater self-esteem in young adults, whereas psychological control (i.e., autonomy-suppressive parenting) showed the inverse (Gong and Wang, 2023). Long-term benefits are also evident; individuals who recall warm, caring parents are more likely to use problem-solving coping strategies and less likely to rely on maladaptive emotional coping in adulthood, even up to 20 years later (Moran et al., 2018). In early adolescence, warm and supportive mothers help their daughters manage conflicts and develop better emotion regulation skills, whereas non-supportive reactions reduce their ability to manage anger and sadness (Berona et al., 2023). Collectively, these studies emphasize how autonomy-supportive and warm parenting can foster optimal well-being by promoting adaptive emotional responses in individuals across their lifetime.

### 1.3 From reactive to proactive: regulating positive emotions via emotion crafting

As mentioned earlier, parenting practices are crucial in developing their offspring’s emotion regulation abilities (e.g., Morris et al., 2007, 2017). However, research to date has predominantly focused on the regulation of negative emotions, including research on parental autonomy support and warmth (e.g., Brenning et al., 2015). Focusing on the regulation of positive emotions might offer several advantages. For example, positive emotions have been linked to key well-being indicators, such as psychological growth and better mental and physical health (Quoidbach et al., 2015; Reitsema et al., 2022). They can also foster the development of lasting social, psychological, and cognitive assets (see PERMA model; Turner et al., 2023), even when controlling for the absence of negative emotions (Pressman and Cohen, 2005).

Several theoretical perspectives underscore the importance of cultivating positive emotions. For example, self-determination theory (Deci and Ryan, 2012) emphasizes that positive affect arises when individuals’ basic psychological needs (i.e., autonomy, competence, relatedness) are satisfied. Self-determination theory views humans as inherently oriented toward growth, authenticity, and proactive engagement with their environment. In a complementary way, the broaden-and-build theory of positive affect (Fredrickson, 2001) posits that positive emotions broaden one’s thought–action repertoire and build enduring resources. These frameworks, alongside perspectives from philosophy and neuroscience, suggest that positive emotions can be actively cultivated through practices like engaging activities, social connection, and meaningful goal pursuit (Larsen et al., 2025).

Promoting positive emotion regulation may be especially beneficial for mental health. One well-established example is savoring, referring to individuals’ ability to notice, value, and enhance positive emotions (Bryant, 2003), which is linked to higher happiness, optimism, and self-esteem (Bryant, 2003), as well as greater life satisfaction and reduced depressive symptoms (Quoidbach et al., 2015). However, savoring is typically reactive, occurring in response to already-present positive emotions, such as enjoying the moment or reminiscing about a past success, rather than proactively generating such experiences (see also Cullen et al., 2024). More broadly, emotion regulation research has largely focused on reactive strategies, those used in response to emotional stimuli (Martins-Klein et al., 2020), such as going for a walk after a stressful conflict. Yet emotion regulation can also be initiated proactively, before an emotional stimulus occurs. For example, a student might plan a movie night with friends the day before an exam, knowing that it will foster positive emotions through social connection. Recent work emphasizes the importance of these proactive strategies, which are implemented before emotions are triggered (Martins-Klein et al., 2020).

The concept of emotion crafting (EC) has been recently introduced to capture individuals’ deliberate efforts to create opportunities for positive emotions in daily life (Van der Kaap-Deeder et al., 2023). EC refers to intentionally initiating, maintaining, or enhancing positive emotions (Van der Kaap-Deeder et al., 2023). EC involves two components: EC awareness, which is the awareness of activities, social situations, or contexts that elicit positive emotions; and EC action, the deliberate pursuit of positive emotions through behavior. To illustrate, knowing that spending time with friends and cooking brings joy (awareness) enables individuals to invite friends over for dinner (action), thereby positively shaping their emotional experiences. Hence, EC differentiates itself from the majority of research on emotion regulation, by (1) holding a proactive stance regarding emotion regulation, and (2) changing the focal point from negative to positive emotions.

Previous research showed that EC awareness predicts EC action, which in turn is associated with more positive emotions (Van der Kaap-Deeder et al., 2023), and with higher well-being and fewer anxiety and depressive symptoms. EC showed only weak to moderate associations with adaptive emotion regulation measures, such as adaptive cognitive emotion regulation, cognitive reappraisal, and integrative emotion regulation (*r* = 0.30 to 0.49), suggesting that it represents a related but distinct process. Furthermore, daily EC awareness and action were associated with higher daily positive affect and with lower negative affect, and thereby with more vitality and fewer depressive symptoms that day (Hernandez Hernandez et al., 2025). These associations between EC and well-being were independent from other emotion regulation measures and persisted across days.

### 1.4 Parenting as antecedents of emotion crafting

Although the benefits of EC for well-being have been supported, less is known about the factors that are associated with individuals’ tendency to engage in EC. Given that autonomy-supportive and warm parenting strengthens adaptive forms of reactive emotion regulation (Brenning et al., 2015; Morris et al., 2017), these parenting practices are also likely to foster EC. Parental autonomy support and warmth have been previously linked to greater mindfulness, which involves an enhanced awareness of one’s experiences (Pepping and Duvenage, 2016; Tan et al., 2024) and can offer insight into what makes one feel good, an ability crucial to EC. Autonomy support is particularly conducive to autonomy satisfaction, as it promotes more volitional and proactive functioning (Bülow et al., 2022). Autonomous individuals may be more proactive in regulating their emotions, as they engage in activities willingly rather than out of pressure (Soenens et al., 2017). As autonomy satisfaction has been linked to increased proactive behaviors (Grolnick and Lerner, 2023), this proactive and volitional stance can set the basis for increased EC.

At a broader level, parenting practices such as autonomy support and warmth are best understood not as isolated behaviors but as part of broader family processes. Family systems theory (e.g., Cox and Paley, 1997) and process-oriented models of emotion socialization (e.g., Morris et al., 2007) emphasize that emotion regulation develops in the context of reciprocal, patterned interactions within the family. These early emotional dynamics, including how parents respond to both positive and negative affect, provide scaffolding for children’s own regulation strategies across development. Importantly, research on intergenerational transmission of parenting shows that both positive and negative parenting practices often show modest but reliable continuity from one generation to the next (see Kerr and Capaldi, 2019, for an overview). Positive parenting fosters social competence, educational attainment, and supportive relationships that promote positive parenting in the next generation, whereas negative parenting is transmitted through coercive relationship dynamics and maladjustment, limiting children’s capacity for empathy and emotion regulation and ultimately undermining their caregiving of the next generation (Bos, 2017; Kerr et al., 2009; Neppl et al., 2009; Schofield et al., 2011; Snyder, 2016). This suggests that the autonomy-supportive and warm parenting individuals experience may not only shape their own tendencies for proactive emotion regulation strategies like EC but may also shape how they later parent their own children. In this sense, EC may reflect not just internal capacities but learned relational processes shaped by the family’s emotional climate. Given the well-established benefits of autonomy-supportive and warm parenting for individuals’ well-being via adaptive regulation of negative emotions, it is important to explore whether this association may also be partly driven by proactive strategies such as EC. However, the role of EC in these relations is yet to be explored.

### 1.5 Cultural and parental gender differences

Cultural differences and parental gender have been found to relate to parenting practices, emotion regulation, and well-being (Mesquita et al., 2017; Song et al., 2024; Van Lissa et al., 2019). Our emotions are not solely personal or biological but are also shaped by cultural systems that define how emotions are experienced, interpreted, and regulated (Mesquita et al., 2017). Cultural contexts modify the intensity, frequency, content, meaning, value, and behavioral tendencies associated with emotional experiences and regulation strategies. These differences are functional and meaningful within each culture and contribute to varying levels of well-being (Ma et al., 2018; Mesquita et al., 2017; Song et al., 2024). While most existing research has concentrated on cultural differences in the regulation of negative emotions, relatively few studies have examined how positive emotions are regulated across cultures (e.g., Ma et al., 2018). Exploring how parenting supports the proactive regulation of positive emotions across cultural contexts may therefore offer new insights into the development of well-being in diverse populations.

In terms of parent gender, limited research suggests the distinct roles mothers and fathers may play in emotional development, potentially due to differences in parenting styles and emotional engagement. Mothers are typically more involved in their children’s emotional lives, more likely to address the sources of their emotions, and to promote emotional insight, social–emotional learning, and the expression of emotions (Fivush et al., 2000; Klimes-Dougan et al., 2007). In contrast, fathers often promote risk-taking, resilience, and problem solving and provide exciting emotional stimuli, encouraging children to face challenges and explore their environment (Feldman, 2003; Majdandžić et al., 2014). While some studies highlight similar emotional contributions from both parents (Jaffe et al., 2010), these potentially distinct parenting patterns may shape how young individuals approach emotional exploration and develop strategies for seeking positive experiences. Nevertheless, with much of the current literature emphasizing the reactive regulation of negative emotions, it remains unclear whether mothers’ and fathers’ parenting practices, such as autonomy support and warmth, differentially support proactive forms of positive emotion regulation, such as EC.

### 1.6 Present study

As preregistered (see OSF: https://osf.io/vw6ax), we expected perceived parental autonomy support and warmth to relate to higher EC awareness (Hypothesis 1), which in turn would relate to greater EC action (Hypothesis 2). EC action was then expected to relate to higher levels of resilience and flourishing (Hypothesis 3), two important indicators of youth well-being (Dawson and Pooley, 2013; Richard-Sephton et al., 2024). Thus, we hypothesized a two-step mediation model, where EC awareness and EC action mediate the relation between parenting and well-being (Hypothesis 4), which was expected to remain significant even after controlling for savoring beliefs (Hypothesis 5). In addition, we included gender, education status, marital status, and employment status as covariates in our main models (when MANOVAs indicated significant group differences), as previous research suggests that well-being and EC can vary by participant demographics (e.g., Van der Kaap-Deeder et al., 2023).

We included participants from two Western European countries, Norway and Germany, without specific hypotheses about country effects. Including both countries enabled us to explore potential cross-national differences in the associations between parenting, EC, and well-being. In addition, we collected data on participants’ perceptions of both their mother’s and father’s parenting, which allowed us to explore potential differences based on parent gender. Therefore, in an exploratory manner, we aimed to examine group differences based on (1) country of residence (Norway or Germany), and (2) parent gender (mother or father) in our models (i.e., parenting → EC → well-being).

## 2 Materials and methods

### 2.1 Participants

The sample consisted of 254 young adults aged between 18 and 29 years (*M* = 22.70, *SD* = 2.07), predominantly women (69.3, and 28.3% men, 1.6% non-binary, and 0.8% chose not to disclose their gender). Most of them lived in Norway (*N* = 162, 63.8%) and about one third in Germany (*N* = 92, 36.2%). The sample was diverse in terms of educational background: 2.8% had less than a high school education, 53.7% were high school graduates or had an equivalent qualification, and 7.7% had completed trade, technical, or vocational training. A further 26.4% held a bachelor’s degree, and 9.3% had obtained a master’s degree. Most participants were students at the time of the study (76.4%). Regarding relationship status, 57.1% were single, 41.7% were in a relationship but not married, and 1.2% were married. Employment status varied, with 47.6% working part-time, 20.9% employed full-time, and 31.5% not currently employed.

### 2.2 Procedure

Data for this cross-sectional study were collected in Norway (November 2022) and Germany (February–March 2023) by trained bachelor’s and master’s students as part of their thesis work. The inclusion of both countries was planned from the outset; however, the resulting sample included a higher proportion of participants from Norway (63.8%), which reflects differences in recruitment reach rather than intentional oversampling. Inclusion criteria required participants to be aged 18–29. Recruitment was conducted through convenience and snowball sampling, primarily via students’ social networks, social media (through personal posts and messages on Instagram, Facebook, and WhatsApp). Online questionnaires were completed anonymously in Norwegian and German, corresponding to the participants’ country of residence. Informed consent was obtained, and The International Test Commission (ITC) guidelines were followed for translating questionnaires not available in Norwegian or German. The Norwegian data collection was guided by the Norwegian Agency for Shared Services in Education and Research (Sikt; reference number 692256), while the German procedure was approved by the Ethics Review Committee of Utrecht University (reference number 22-2075).

### 2.3 Measures

Internal consistency for all measures was assessed using Cronbach’s *α* (Cronbach, 1951) and McDonald’s *ω* (McDonald, 1999), with values ≥ 0.70 considered acceptable, ≥ 0.80 considered good, and ≥ 0.90 considered excellent (Nunnally and Bernstein, 1994).

#### 2.3.1 Perceptions of Parents Scale

The Perception of Parents Scale (Robbins, 1994) measures individuals’ perceptions of parental autonomy support with nine items, such as “My mother/father, whenever possible, allows me to choose what to do,” and parental warmth with six items, such as “My mother/father clearly conveys her/his love for me.” Each item is rated on a 7-point Likert scale (1 = not at all true, 7 = very true). The scale was administered separately for perceptions of one’s mother and father, resulting in a total of 30 items. Both the autonomy support (*α* = 0.89, *ω* = 0.87) and warmth (α = 0.87, ω = 0.86) subscales exhibited good reliability.

#### 2.3.2 Emotion Crafting Scale

The Emotion Crafting Scale (Van der Kaap-Deeder et al., 2023) assesses EC and thereby refers to individuals’ awareness of positive-emotion inducing situations and their proactive efforts to strengthen positive emotions. The scale comprises 12 items, each rated on a 5-point Likert scale (1 = strongly disagree, 5 = strongly agree). The scale is structured around two components: awareness (four items; e.g., “I know well which activities make me feel good.”) and action (eight items; e.g., “I deliberately do as many activities as possible which make me feel good.”). Reliability was good for the awareness subscale (α = 0.82, ω = 0.80) and acceptable for the action subscale (α = 0.71, ω = 0.71).

#### 2.3.3 Brief Resilience Scale

The Brief Resilience Scale (Smith et al., 2008) measures psychological resilience – the ability to recover from stress. This unidimensional scale comprises six items (e.g., “I tend to bounce back quickly after hard times.”), each rated on a 5-point Likert scale (1 = strongly disagree, 5 = strongly agree). The scale exhibited good reliability (α = 0.87, ω = 0.87).

#### 2.3.4 Flourishing Scale

The Flourishing Scale (Diener et al., 2010) measures psychological flourishing, in other words, individuals’ social and psychological prosperity. The scale is unidimensional and comprises eight items (e.g., “I am engaged and interested in my daily activities.”), each rated on a 7-point Likert scale (1 = strongly disagree, 7 = strongly agree). The scale displayed good reliability (α = 0.88, ω = 0.88).

#### 2.3.5 Savoring Beliefs Inventory

The Savoring Beliefs Inventory (Bryant, 2003) measures individuals’ beliefs about their ability to notice, value, and enhance positive emotions. Employed as a unidimensional scale, the scale comprises 24 items (e.g., “I know how to make the most of a good time.”), each rated on a 7-point Likert scale (1 = strongly disagree, 7 = strongly agree). The scale exhibited strong reliability (α = 0.93, ω = 0.93).

### 2.4 Preregistered analysis plan

We conducted the analyses in line with our preregistration (see OSF: https://osf.io/vw6ax). We first obtained descriptive values of and correlations between the study variables. Estimates of effects of the demographic variables on the dependent variables (through (M) ANOVAs) allowed us to control for such predictors in our main models. Gender was reduced to a binary variable (female and male), due to insufficient representation in other categories (2.4% in total).

Subsequently, we fitted two separate structural serial mediation models to examine whether EC (partly) mediated the link between perceived parental autonomy support or perceived parental warmth and well-being (i.e., resilience and flourishing). Given the high correlation between the parenting variables (*r* = 0.82), they were analyzed separately to avoid multicollinearity and clarify each predictor’s contribution. EC was modeled as a two-step serial mediation, with EC awareness preceding EC action, in line with the theory (Van der Kaap-Deeder et al., 2023).

Third, sensitivity analyses were conducted to assess the robustness of our findings by re-estimating the final models after (1) excluding outliers and careless respondents, (2) adding gender as a control variable, and (3) adding savoring beliefs as a control variable in the relation between parenting and well-being. Univariate outliers were identified using standardized Z-scores exceeding ± 3.29, while multivariate outliers were detected using Mahalanobis distances with a critical chi-square value (*p* < 0.001; Tabachnick and Fidell, 2013). We identified careless responders using five different analyses: long-string, even-odd consistency, intra-individual response variability, psychometric synonyms, and psychometric antonyms (Arthur et al., 2021). Participants flagged by at least three of these methods were classified as careless respondents.

Fourth, we examined, in an explorative manner, whether the overall models (i.e., parenting → EC → well-being) differed based on country of residence (Norway vs. Germany) and parent gender (maternal vs. paternal parenting), excluding control variables for simplicity. We examined country differences using multigroup analyses in which an unconstrained model with all path coefficients varying freely is tested against a model with constraints, where all coefficients were held equal across both groups. Model fit differences were assessed using the chi-square difference test (Δχ^2^) and the change in comparative fit index (ΔCFI), where a non-significant Δχ^2^ (*p* > 0.05) and ΔCFI < 0.01 indicate that constraining parameters did not significantly worsen model fit (Cheung and Rensvold, 2002). If the model fit was significantly different, this suggested that the relations between variables varied between Norwegian and German participants. In such cases, we applied a stepwise partial constraint approach, systematically testing which paths could be constrained without significantly affecting model fit, helping to identify the specific associations that differed across groups.

To test whether maternal and paternal parenting had distinct associations in our final models, we conducted equality constraint testing separately for each parenting model (autonomy support and warmth). Instead of using a composite parental score as in our previous models (e.g., parental warmth), we analyzed maternal and paternal parenting separately (e.g., maternal warmth and paternal warmth). For each model, we compared an unconstrained version (freely varying coefficients) with a constrained version (equal coefficients for all parenting paths), and applied a stepwise partial constraint approach where necessary, following the same procedure as before.

These analyses were performed using SPSS version 29 (IBM Corp, 2021) and RStudio (Posit Team, 2023). The missing data (maternal parenting 3.5%, paternal parenting 5.5%, EC 0%, resilience 4.3%, flourishing 5.5%, savoring 4.3%) were missing completely at random, as indicated by the normed χ^2^/*df* value of 1.12 (1555.32/1394), which is below the recommended threshold of 2.00 (Ullman, 2001). Therefore, the Full Information Maximum Likelihood (FIML) function was used to handle missing data. Correlations were classified as small (*r* = 0.10), medium (*r* = 0.30), and large (*r* = 0.50), following Cohen’s guidelines (Cohen 1988). Model fit was assessed using the χ^2^ test, CFI, TLI, SRMR, and RMSEA. Acceptable-to-good fit criteria were indicated by χ^2^/*df* ≤ 3, CFI/TLI ≥ 0.90, SRMR ≤ 0.10, and RMSEA ≤ 0.08, following the guidelines of Browne and Cudeck (1992), Hu and Bentler (1999), and Kline (2015). All tests adhered to a *p* < 0.05 significance level.

### 2.5 Deviation from the preregistration

The sample size in our preregistration was specified as 267 young adults. However, 13 participants were above the age of 29, exceeding our age cut-off criteria (18–29). Therefore, we included 254 participants. Additionally, instead of the preregistered exploratory moderated mediation analyses to test for differences based on country and parent gender, we conducted multigroup modeling to simplify interpretation and ensure robust comparisons.

## 3 Results

### 3.1 Preliminary analyses

EC awareness and action showed positive correlations with perceived parental autonomy support and warmth, well-being, and savoring beliefs, see Table 1. EC awareness showed medium associations with parenting, flourishing, and savoring, while EC action showed small associations with parenting and resilience, and moderate associations with flourishing and savoring. Additionally, the EC variables were strongly correlated with each other, as were the well-being variables and the parenting variables.

**Table 1:** Descriptives of and correlations between the study variables.

Variables	*M*	*SD*	1	2	3	4	5	6
1. Autonomy support	5.46	0.93						
2. Warmth	6.01	0.88	0.82					
3. EC awareness	4.35	0.59	0.37	0.40				
4. EC action	3.95	0.52	0.16	0.21	0.53			
5. Resilience	3.35	0.82	0.21	0.22	0.25	0.16		
6. Flourishing	5.71	0.82	0.37	0.37	0.41	0.38	0.46	
7. Savoring beliefs	5.26	0.87	0.37	0.32	0.46	0.61	0.46	0.58

Perceived parental autonomy support and warmth, see method section; EC = emotion crafting. Correlations r = 0.16 were significant at *p* < 0.05 and the rest at *p* < 0.001.

The first MANOVA, focusing on EC awareness, EC action, and savoring beliefs, showed no significant differences due to country, education, marital status, or employment status in the combined dependent variables (*F* values ranging between 0.47 and 1.63, *p*s > 0.05). However, gender had a significant multivariate effect (*F*_(3, 203)_ = 4.39, *p* < 0.05, η^2^ = 0.06), and our follow-up ANOVA indicated that EC action was higher for women than men (*d* = 0.25, *M* = 4.03, *SD* = 0.50, versus *M* = 3.78, *SD* = 0.54; *F*_(1, 246)_ = 12.31, *p* < 0.001, η^2^ = 0.05). Similarly, savoring beliefs were stronger for women than men (*d* = 0.27, *M* = 5.37, *SD* = 0.83 versus *M* = 5.10, *SD* = 0.92; *F*_(1, 222)_ = 4.18, *p* < 0.05, η^2^ = 0.02). EC awareness showed no gender differences (*F*_(1, 246)_ = 0.75, *p* > 0.05).

The second MANOVA revealed no significant differences in resilience and flourishing depending on the demographics (*F* values ranging between 0.90 and 2.02, *p*s > 0.05), except for gender (*F*_(2, 214)_ = 5.19, *p* < 0.05, η^2^ = 0.05); our univariate ANOVA revealed that resilience was higher among men than women (Cohen’s *d* = 0.28, *M* = 3.56, *SD* = 0.81) than women (*M* = 3.28, *SD* = 0.81; *F*_(1, 235)_ = 5.85, *p* < 0.05, η^2^ = 0.03). No gender differences in flourishing were reported (*F*_(1, 232)_ = 0.26, *p* > 0.05). Overall, since the preliminary analyses indicated that EC action, savoring beliefs, and resilience significantly differed by gender, paths from gender to these variables were added in the subsequent analyses to control for its effect.

### 3.2 Primary analyses

We first examined the structural models for parental autonomy support and warmth, finding all hypothesized paths to be significant. Subsequently, we tested the significance of non-hypothesized direct effects, such as from autonomy support to EC action. The final model comprised all significant paths and showed marginally acceptable fit for both the autonomy support model (χ*^2^* (6) = 18.91, *p* < 0.05, CFI = 0.95, TLI = 0.88, RMSEA = 0.10, SRMR = 0.06) and the warmth model (χ*^2^* (6) = 19.68, *p* < 0.05, CFI = 0.95, TLI = 0.87, RMSEA = 0.10, SRMR = 0.06).

**Figure 1:**
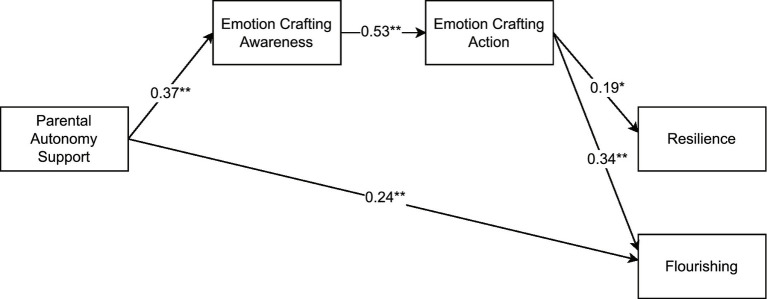
From parental autonomy support to well-being via emotion crafting – final model. The effects of gender on emotion crafting action and resilience were included in the model but are not displayed for reasons of clarity. *p < 0.01; **p < 0.001.

**Figure 2:**
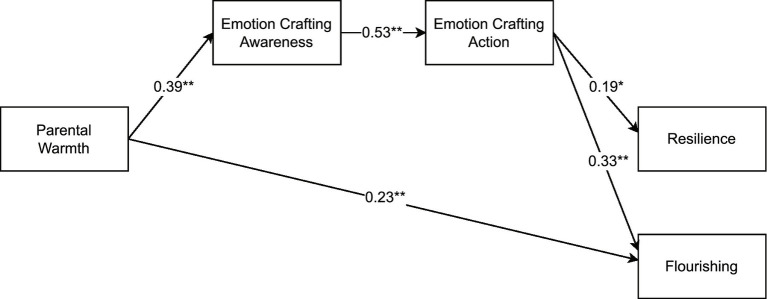
From parental warmth to well-being via emotion crafting – final model. The effects of gender on emotion crafting action and resilience were included in the model but are not displayed for reasons of clarity. *p < 0.01; **p < 0.001.

In the final models, both autonomy support (see Figure 1) and warmth (see Figure 2) were significantly and positively related to EC awareness, which in turn related positively to EC action, which predicted resilience and flourishing (serial mediation). Gender also related to both EC action (βs = 0.19, *p*s < 0.01) and resilience (βs = −0.19, *p*s < 0.01), such that women reported more EC action and men reported more resilience, on average. Only two non-hypothesized paths were retained, with both autonomy support and warmth directly and positively relating to flourishing.

The indirect effect of autonomy support on resilience and flourishing via EC awareness and EC action indicated full mediation for resilience (β = 0.04, *p* < 0.01) and partial mediation for flourishing (β = 0.07, *p* < 0.001). Similarly, the indirect effect of warmth on resilience and flourishing via EC awareness and EC action indicated full mediation for resilience (β = 0.04, *p* < 0.01) and partial mediation for flourishing (β = 0.07, *p* < 0.001). These models (i.e., focusing on autonomy support or warmth) explained 13.3 and 15.2% of the variance in EC awareness, 30.8 and 30.7% in EC action, 5.9 and 6% in resilience, and 20.6 and 19.2% in flourishing, respectively.

#### 3.2.1 Sensitivity analyses

A total of 32 cases were identified as outliers or careless responders (12.6%). The final models were tested with and without these cases which yielded virtually identical results. Therefore, these cases were retained in the dataset. Subsequently, to control for savoring beliefs, paths from autonomy support and warmth to savoring beliefs and from savoring beliefs to resilience and flourishing were added to both final models. These models displayed an excellent fit for autonomy support (χ*^2^* (6) = 10.39, *p* > 0.05, CFI = 0.99, TLI = 0.97, RMSEA = 0.06, SRMR = 0.03) and warmth (χ*^2^* (6) = 10.61, *p* > 0.05, CFI = 0.99, TLI = 0.96, RMSEA = 0.06, SRMR = 0.03). Both autonomy support and warmth related to EC awareness (β_autonomy_ = 0.37, β_warmth_ = 0.39, *p*s < 0.001), which then related to EC action (βs = 0.53, *p*s < 0.001). EC action, however, did not anymore relate to resilience or flourishing (*p*s > 0.05). Savoring beliefs were predicted by both parenting constructs (β_autonomy_ = 0.37, β_warmth_ = 0.31, *p*s < 0.001), and were in turn linked to resilience (βs = 0.53, *p*s < 0.001) and flourishing (β_autonomy_ = 0.45, β_warmth_ = 0.46, *p*s < 0.001). Additionally, direct paths from autonomy support and warmth to flourishing were retained in the final model (βs = 0.18, *p*s = 0.001). Finally, gender related to EC action (βs = 0.19, *p*s = 0.001), resilience (βs = −0.19, *p*s = 0.001), and savoring beliefs (βs = 0.12, *p*s < 0.05).

### 3.3 Exploratory analyses

#### 3.3.1 Country

We conducted a multigroup comparison to examine whether the observed associations between positive parenting practices, EC, and well-being would be similar for young adults living in Norway and Germany. The constrained autonomy support model fitted the data equally well as the unconstrained model (Δχ^2^ (5) = 9.53, *p* > 0.05 and ΔCFI = 0.018), which indicates that the relations illustrated in Figure 1 were consistent among young adults from both countries. For the warmth model, the unconstrained model fit significantly better than the fully constrained model (Δχ^2^ (5) = 16.89, *p* < 0.01 and ΔCFI = 0.04), but did not differ significantly from the partially constrained model (Δχ^2^ (3) = 4.92, *p* > 0.05 and ΔCFI = 0.007). More specifically, in the partially constrained model, warmth was significantly associated with EC awareness in Norway (β = 0.51, *p* < 0.001) but not in Germany (β = 0.17, *p* > 0.05). Similarly, EC action was linked to flourishing in Norway (β = 0.44, *p* < 0.001) but not in Germany (β = 0.12, *p* > 0.05). Other associations remained similar across groups.

#### 3.3.2 Parent gender

We conducted equality constrained testing in both final models, using separate maternal and paternal autonomy support and warmth scores. For both the autonomy support and warmth models, the unconstrained models fit significantly better than the fully constrained models (autonomy support: Δχ^2^ (2) = 6.59, *p* < 0.05, ΔCFI = 0.018 and warmth: Δχ^2^ (2) = 9.80, *p* < 0.01, ΔCFI = 0.03), but did not differ significantly from the partially constrained models (autonomy support: Δχ^2^ (1) = 0.15, *p* > 0.05, ΔCFI = −0.003 and warmth: Δχ^2^ (1) = 0.33, *p* > 0.05, ΔCFI = −0.003). In the partially constrained models, the paths from maternal and paternal parenting (autonomy support and warmth) to flourishing were constrained to be equal, indicating that maternal and paternal parenting had comparable associations with flourishing. In contrast, the paths from maternal and paternal parenting to EC awareness remained freely estimated, as constraining these paths significantly worsened model fits, indicating that maternal and paternal parenting contribute differently to EC awareness. In both unconstrained models, maternal parenting was more strongly associated with EC awareness (β_autonomy_ = 0.34, β_warmth_ = 0.38, *p* < 0.001) compared to paternal parenting, whose associations were positive but non-significant (β_autonomy_ = 0.09, β_warmth_ = 0.06, *p* > 0.05).

## 4 Discussion

Parenting practices have long-lasting implications for individuals’ health, relationships, and psychological adjustment, with offspring’s emotion regulation being a key mechanism (Morris et al., 2021; Yu et al., 2019). However, while much of the literature has emphasized how parenting relates to the regulation of negative emotions, less is known about how positive parenting encourages proactive strategies to strengthen positive emotions, a process increasingly recognized as central to well-being (Quoidbach et al., 2015; Van der Kaap-Deeder et al., 2023). We therefore focused on EC as a proactive strategy to deliberately strengthen positive emotions and examined its mediating role in the relations from perceived parental autonomy support and warmth to youth well-being. By doing so, the present study offers new insights into the possible pathways linking positive parenting with young adults well-being.

Our analyses relied on data from 254 young adults in Norway and Germany. We found that both parental autonomy support and warmth were significantly associated with greater EC awareness, which in turn related to higher EC action, and ultimately to greater resilience and flourishing (serial mediation). However, the associations between EC action and well-being were not significant after accounting for savoring beliefs. Finally, we observed cultural and parent gender differences in our main models, which are detailed in the following sections.

### 4.1 From parental autonomy support and warmth to emotion crafting

The present findings indicated that both perceived parental autonomy support and warmth were associated with greater EC awareness, in line with Hypothesis 1. This suggests that individuals exposed to autonomy-supportive and warm parenting were more aware of what makes them feel good. With these results, our study is among the first to link positive parenting practices to individuals’ positive emotion regulation, and specifically their awareness of positive-emotion-inducing contexts. This aligns with previous research showing that positive parenting is linked to higher mindfulness, which is characterized by awareness of one’s internal experiences, such as thoughts and bodily sensations (Pepping and Duvenage, 2016; Tan et al., 2024), as well as more adaptive emotion regulation strategies (Brenning et al., 2015; Jaffe et al., 2010). Autonomy-supportive parenting has been previously associated with greater emotion integration, characterized by openness, acceptance and curiosity toward negative emotions (Brenning et al., 2015). Our findings suggest that this may extend to positive emotions via EC, which allows individuals to become more aware of situations that elicit positive feelings.

Parental warmth also plays a crucial role in helping children recognize and foster positive emotions (Davidov and Grusec, 2006; Zhou et al., 2002) and has been shown to relate to more adaptive and fewer maladaptive emotion regulation strategies (Jaffe et al., 2010; Tani et al., 2018). Warm parent–child interactions may encourage offspring to become more attuned to situations that induce positive emotions, potentially explaining why parental warmth was significantly associated with EC awareness in our study.

Our findings further showed EC awareness to positively relate to EC action, in line with Hypothesis 2 and previous research (e.g., Hernandez Hernandez et al., 2025). Van der Kaap-Deeder et al. (2023) argued that EC awareness is a necessary prerequisite for engaging in EC action, as individuals first need to know what makes them feel good, before they can take actions to strengthen their current or future positive emotions (also see Gross, 2015). The stages of change model in psychotherapy also highlights awareness as a fundamental first step in moving toward behavioral change (Krebs et al., 2018). Similar to how individuals progress through the action stage in therapy, those with higher awareness of positive emotion eliciting contexts (EC awareness) may be more prepared and motivated to proactively engage in behaviors aimed at cultivating those emotions (EC action).

Overall, the relation between positive parenting practices and EC reinforces the idea that emotion regulation is not solely a matter of time and maturation, but an interpersonal process (Ryan et al., 2016, p. 409). This process begins with caregivers’ regulation of their child’s emotions, shaped by the adaptiveness of their parenting strategies; and their children learn from this relationship and ideally develop the ability to regulate their own emotions autonomously and proactively (Sigelman and Rider, 2014). In this context, our findings suggest that young adults who perceive their parents as autonomy-supportive and warm are more likely to be aware of their positive emotional experiences and transform this awareness into proactive, volitional action, to regulate their positive emotions, which closely aligns with the definition of adaptive emotion regulation in the self-determination theory (Ryan et al., 2016, p. 407).

These findings are particularly meaningful in the context of young adulthood, a developmental stage marked by ongoing identity exploration, increased autonomy, and prolonged transitions in education, employment, and relationships (Arnett, 2000). In particular, romantic relationships have long been recognized as central to overall adjustment and well-being during this period, with young adults’ reliance on social support for emotion regulation peaking during this stage compared to both earlier and later life stages (Zimmermann and Iwanski, 2014). Yet long-term committed partnerships and independent living are often delayed into the thirties, particularly in Western industrialized contexts (Shulman and Connolly, 2013). Consequently, alongside support from close romantic relationships, young adults may continue to rely on their parents for emotional guidance and support (Nelson, 2022), making parental autonomy support and warmth especially relevant for their well-being. EC, as a proactive and volitional strategy, may serve as a crucial regulatory skill during this transitional phase, enabling young adults to navigate uncertainty and promote well-being through self-initiated positive emotional experiences.

### 4.2 The role of individuals’ emotion crafting in resilience and flourishing

EC action was associated with well-being both in terms of resilience and flourishing, in line with Hypothesis 3. According to Fredrickson (2001), positive emotions are the markers of flourishing. As the aim of EC action is to strengthen positive emotions, the observed association between these constructs is consistent with theoretical expectations. With regard to resilience, a smaller but still positive link was found with EC action. Given that resilience focuses on individuals’ ability to bounce back from adversity, EC action might play a more indirect role. This is consistent with the “undoing hypothesis” (Fredrickson and Levenson, 1998), which states that positive emotions can undo the aftereffects of negative emotions. Thus, EC action might build individuals’ resilience via counteracting negative emotions through positive ones.

Additionally, the relation between the positive parenting practices and well-being was sequentially mediated by the awareness and action components of EC, supporting Hypothesis 4. Participants who perceived that their volitional functioning was nurtured by emotionally warm and responsive parents were more likely to understand what makes them feel good and take proactive steps to seek out such experiences. In turn, this related to higher levels of flourishing and resilience. This aligns with previous research showing that these parenting practices promote more adaptive emotion regulation strategies (Jaffe et al., 2010), which in turn, are linked to greater well-being (Brenning et al., 2015).

Despite these significant relations, the effects of EC action on resilience and flourishing were no longer significant when savoring beliefs were added to the models, contrary to Hypothesis 5. This pattern may be explained by several factors. First, this study is the first to examine the direct association between EC action and well-being without including positive affect as a mediator. It is possible that EC action primarily contributes to well-being by increasing positive affect, rather than playing a direct role, a possibility that should be explored in future research. Second, controlling for savoring beliefs may have led to a suppression effect, aligning with Lynam et al.’s (2006) view on the interpretive challenges of partialling out highly related constructs. Similar findings on parenting and well-being emerged before, where controlling for a related variable changed or even reversed the direction of an effect (Bhargava et al., 2014). In our case, when controlling for savoring beliefs, a construct conceptually similar to EC action in its aim to enhance positive emotions, the shared variance between the two was removed. This likely left a residualized version of EC action that no longer fully reflects its theoretical construct, potentially capturing less adaptive forms. Savoring typically involves appreciating existing positive experiences (Livingstone and Srivastava, 2012), whereas EC action entails proactively seeking and generating them. Since proactive emotion regulation strategies have been studied less extensively than response-focused ones (e.g., savoring, reappraisal), their unique benefits may be harder to detect, especially when reactive strategies like savoring are already in use. In such cases, the added value of EC action may be reduced. As this may be the first study to examine this distinction, further research is needed to clarify the differential effects of proactive and reactive strategies on well-being.

### 4.3 Group differences based on country of residence and parent gender

We explored whether the associations between parenting and well-being, mediated by EC, varied by country of residence and parent gender using multigroup modeling. The associations between autonomy support and well-being were consistent across countries but youth in Norway showed stronger associations between parental warmth and EC awareness than German youth, and also between EC action and flourishing. This could indicate that Norwegian youth, compared to German youth, may respond more sensitively to parental warmth in developing awareness of positive emotional contexts, and are more likely to take action based on this awareness, which in turn contributes to higher flourishing. Although Norway and Germany are often viewed as culturally similar and highly individualistic Western societies (Hofstede et al., 2010), Norwegian young adults typically leave their parental home at an earlier age than their German peers (Billari and Liefbroer, 2010). Parental warmth may therefore play a particularly salient role in supporting Norwegian youths’ proactive emotion regulation efforts and well-being as they navigate the transition to independent living. This interpretation aligns with prior research showing that parental warmth remains protective in young adulthood (Fang et al., 2024), and that cultural norms can shape the strength of its associations with youth outcomes during this developmental period (Chung et al., 2009). Cross-cultural studies on parenting typically compare more distinct cultural groups, such as individualistic vs. collectivistic cultures (e.g., Germany and U. S. versus Spain and Brazil; Garcia et al., 2019). Our results might offer some insights into subtler variations within individualistic cultures, but must be interpreted with caution due to the small sample size, especially for Germany (*N* = 92).

In terms of parent gender, we found that the positive relations between maternal parenting and EC awareness were stronger than the relations for paternal parenting. Previous research on parents’ gender and children’s or young adults’ emotion regulation provides mixed findings, although evidence supporting stronger maternal links tends to dominate. Some studies suggest that both parents contribute similarly to adaptive emotion regulation (Jaffe et al., 2010), while others emphasize the stronger role of mothers, reporting weaker associations for paternal parenting (Davidov and Grusec, 2006). One recent meta-analysis showed that adolescents’ emotion regulation thrived when positive parenting (e.g., parental warmth) was exhibited by mothers, and negative practices including behavioral and psychological control by fathers were reduced (Van Lissa et al., 2019). However, most studies either do not explicitly compare the unique effects of both parents or focus solely on mothers (Bariola et al., 2011). Additionally, the majority of research examines negative emotion regulation, limiting our understanding of the unique contributions of mothers’ and fathers’ positive parenting practices to their offspring’s positive emotion regulation.

One possible explanation for the stronger maternal associations is that mothers, compared to fathers, are typically more engaged in their children’s emotional lives, more likely to discuss the origins of their children’s emotions, and to encourage emotional exploration, socialization, and emotion expression (Fivush et al., 2000; Klimes-Dougan et al., 2007). In line with their autonomy-supportive and warm practices, mothers may also promote the exploration and expression of positive emotions. This could explain why our results showed that positive maternal practices were more strongly associated with young adults’ EC awareness than paternal practices.

### 4.4 Strengths, limitations, practical implications, and suggestions for future research

This study had several strengths, such as examining for the first time possible antecedents of EC, including young adults from two countries, incorporating perceived parenting practices related to both mothers and fathers, and preregistering the hypotheses and analyses. Nonetheless, these findings should be interpreted in light of some limitations. First, our sample consisted mostly of female students. Further studies among more diverse and clinical samples are encouraged. Second, over 60% of our sample was from Norway. Although the overall sample size exceeds the recommended minimum for path analysis (*N* ≥ 200; Kline, 2015), this imbalance reduces power in our multi-group comparisons, as power is mainly driven by the smaller group (Yoon and Lai, 2018). Thus, country-related findings should be interpreted with caution and considered exploratory.

Third, this study relied solely on self-reports. Although previous research has shown that perceived parenting is particularly predictive of psychological functioning (Korelitz and Garber, 2016), future studies could benefit from also incorporating parent reports, as well as more objective well-being measures such as behavioral assessments or physiological data. Moreover, the correlational and cross-sectional nature of the study limits our ability to infer causality. Although we proposed that parental autonomy support and warmth can support young adults’ well-being through EC, it is also plausible that youth with higher levels of resilience and flourishing may perceive their parents more positively or may engage more frequently in EC. While theoretical models and previous research support our proposed direction of variable relations (e.g., Brenning et al., 2015; Grolnick and Lerner, 2023), mediation analyses with cross-sectional data cannot establish temporal precedence. Future longitudinal or experimental studies are essential to test the causal ordering of the associations between parenting, EC, and well-being.

Finally, as noted by Hernandez Hernandez et al. (2025), exploring the effects of EC across more diverse cultures is crucial. For instance, in East Asian cultures, there is a greater emphasis on balancing both positive and negative emotions rather than focusing solely on enhancing positive ones (Miyamoto et al., 2017). This raises the question of whether EC would produce similar outcomes in such cultural contexts.

Beyond theoretical contributions, our findings also offer practical implications for parenting interventions, emotional education, well-being, and resilience-building programs to support youths’ emotional development. Given that EC is associated with positive parenting practices, programs targeting parents may benefit from emphasizing ways to foster positive emotional exploration and the intentional pursuit of positive experiences. For instance, parents can support EC engagement by modeling positive emotion regulation, this by encouraging youth to identify and pursue enjoyable activities, creating space for reflection, validating their emotional goals, and promoting small proactive ways to boost daily well-being (e.g., nature walks, hobbies, or social time). Such strategies may help young adults develop internal resources to support long-term flourishing.

Although our results showed that maternal parenting was more strongly related to EC awareness than paternal parenting, existing literature suggests that fathers may play a distinct role in shaping their offspring’s emotional experiences, such as by promoting risk-taking, encouraging exploration, and providing exciting emotional stimuli (Feldman, 2003; Majdandžić et al., 2014). Fathers may foster EC through pathways that differ from those typically associated with maternal support. Rather than emphasizing emotional discussion, fathers might encourage autonomy, exploration, and engagement in challenging or novel situations. These interactions may help young adults discover what makes them feel good and encourage them to take the initiative to try new things, thereby supporting the development of EC awareness and action. Future research and interventions may benefit from exploring how fathers can uniquely support EC development.

## 5 Conclusion

This study contributes to the growing field of proactive emotion regulation by being the first to examine potential antecedents of EC. Specifically, out findings suggest that parental autonomy support and warmth were associated with higher EC awareness and action, which in turn related to greater flourishing and resilience. When controlling for savoring beliefs, however, the association between EC action and well-being disappeared, suggesting a theoretical overlap. Future studies are needed to explore how these strategies interact and vary across different populations and cultural contexts.

## Data Availability

The raw data supporting the conclusions of this article will be made available by the authors, without undue reservation.

## References

[ref1] Arnett J. J. (2000). Emerging adulthood: a theory of development from the late teens through the twenties. Am. Psychol. 55:469. doi: 10.1037/0003-066X.55.5.469, PMID: 10842426

[ref2] Arthur W. Jr. Hagen E. George F. Jr. (2021). The lazy or dishonest respondent: detection and prevention. Annu. Rev. Organ. Psychol. Organ. Behav. 8, 105–137. doi: 10.1146/annurev-orgpsych-012420-055324

[ref3] Bai S. Repetti R. L. Sperling J. B. (2016). Children’s expressions of positive emotion are sustained by smiling, touching and playing with parents and siblings: a naturalistic observational study of family life. Dev. Psychol. 52:88. doi: 10.1037/a0039854, PMID: 26524382 PMC4695297

[ref4] Barber B. K. Olsen J. E. Shagle S. C. (1994). Associations between parental psychological and behavioural control and youth internalised and externalised behaviours. Child Dev. 65:4. doi: 10.2307/1131309 7956469

[ref5] Bariola E. Gullone E. Hughes E. K. (2011). Child and adolescent emotion regulation: the role of parental emotion regulation and expression. Clin. Child. Fam. Psychol. Rev. 14:2. doi: 10.1007/s10567-011-0092-5 21424275

[ref6] Berona J. Sroka A. W. Gelardi K. L. Guyer A. E. Hipwell A. E. Keenan K. (2023). Maternal socialisation of emotion and the development of emotion regulation in early adolescent girls. Emotion 23, 872–878. doi: 10.1037/emo0001110, PMID: 35939601 PMC9905353

[ref7] Bhargava S. Kassam K. S. Loewenstein G. (2014). A reassessment of the defence of parenthood. Psychol. Sci. 25:1. doi: 10.1177/0956797613503348 24220628

[ref8] Billari F. C. Liefbroer A. C. (2010). Towards a new pattern of transition to adulthood? Adv. Life Course Res. 15, 59–75. doi: 10.1016/j.alcr.2010.10.003, PMID: 41046192

[ref9] Bos P. A. (2017). The endocrinology of human caregiving and its intergenerational transmission. Dev. Psychopathol. 29, 971–999. doi: 10.1017/S0954579416000973, PMID: 27760577

[ref10] Brenning K. Soenens B. Van Petegem S. Vansteenkiste M. (2015). Perceived maternal autonomy support and early adolescent emotion regulation: a longitudinal study. Soc. Dev. 24:3. doi: 10.1111/sode.12107

[ref11] Browne M. W. Cudeck R. (1992). Alternative ways of assessing model fit. Sociol. Methods Res. 21, 230–258. doi: 10.1177/0049124192021002005

[ref12] Bryant F. B. (2003). Savoring beliefs inventory (SBI): a scale for measuring beliefs about savoring. J. Ment. Health 12:2. doi: 10.1080/0963823031000103489

[ref13] Bülow A. Neubauer A. B. Soenens B. Boele S. Denissen J. J. A. Keijsers L. (2022). Universal ingredients to parenting teens: parental warmth and autonomy support promote adolescent well-being in most families. Sci. Rep. 12:16836. doi: 10.1038/s41598-022-21071-0, PMID: 36207448 PMC9546835

[ref14] Butterfield R. D. Silk J. S. Lee K. H. Siegle G. S. Dahl R. E. Forbes E. E.. (2021). Parents still matter! Parental warmth predicts adolescent brain function and anxiety and depressive symptoms two years later. Dev. Psychopathol. 33, 226–239. doi: 10.1017/S0954579419001718, PMID: 32096757 PMC7483163

[ref15] Chen Y. Kubzansky L. D. VanderWeele T. J. (2019). Parental warmth and flourishing in mid-life. Soc. Sci. Med. 220, 65–72. doi: 10.1016/j.socscimed.2018.10.026, PMID: 30396119 PMC6309475

[ref16] Cheung G. W. Rensvold R. B. (2002). Evaluating goodness-of-fit indexes for testing measurement invariance. Struct. Equ. Model. 9:2. doi: 10.1207/S15328007SEM0902_5

[ref9002] Chung W. Y. Chen C. Greenberger E. Heckhausen J. (2009). A cross-ethnic study of adolescents’ depressed mood and the erosion of parental and peer warmth during the transition to young adulthood. J. Res. Adolesc. 19:3. doi: 10.1111/j.1532-7795.2009.00592.x

[ref17] Cohen J. (1988). Statistical power analysis for the behavioural sciences. 2nd Edn. Hillsdale, NJ: Lawrence Erlbaum Associates.

[ref18] Costa S. Cuzzocrea F. Gugliandolo M. C. Larcan R. (2016). Associations between parental psychological control and autonomy support, and psychological outcomes in adolescents: the mediating role of need satisfaction and need frustration. Child Indic. Res. 9:4. doi: 10.1007/s12187-015-9353-z

[ref19] Cox M. J. Paley B. (1997). Families as systems. Annu. Rev. Psychol 48, 243–267. doi: 10.1146/annurev.psych.48.1.243 9046561

[ref20] Cronbach L. J. (1951). Coefficient alpha and the internal structure of tests. Psychometrika 16:3. doi: 10.1007/BF02310555

[ref21] Cullen K. Murphy M. Di Blasi Z. Bryant F. (2024). The effectiveness of savouring interventions on well-being in adult clinical populations: a protocol for a systematic review. PLoS One 19:e0302014. doi: 10.1371/journal.pone.0302014, PMID: 38626110 PMC11020756

[ref22] Davidov M. Grusec J. E. (2006). Untangling the links of parental responsiveness to distress and warmth to child outcomes. Child Dev. 77:44. doi: 10.1111/j.1467-8624.2006.00855.x, PMID: 16460524

[ref23] Dawson M. Pooley J. (2013). Resilience: the role of optimism, perceived parental autonomy support and perceived social support in first-year university students. J. Educ. Train. Stud. 1, 38–49. doi: 10.11114/jets.v1i2.137

[ref24] Deci E. L. Ryan R. M. (2012). “Self-determination theory” in Handb. Theor. Soc. Psychol. eds. Van Lange P. A. M. Kruglanski A. W. Higgins E. T. (London: Sage), 416–436.

[ref25] Diener E. Wirtz D. Tov W. Kim-Prieto C. Choi D. W. Oishi S.. (2010). New well-being measures: short scales to assess flourishing and positive and negative feelings. Soc. Indic. Res. 97, 143–156. doi: 10.1007/s11205-009-9493-y

[ref26] Duineveld J. J. Parker P. D. Ryan R. M. Ciarrochi J. Salmela-Aro K. (2017). The link between perceived maternal and paternal autonomy support and adolescent well-being across three major educational transitions. Dev. Psychol. 53, 1978–1994. doi: 10.1037/dev0000364, PMID: 28805437

[ref27] Fang S. Fosco G. M. Feinberg M. E. (2024). Parental warmth and young adult depression: a comparison of enduring effects and revisionist models. Dev. Psychopathol. 36, 1849–1862. doi: 10.1017/S0954579423001207, PMID: 37752730

[ref28] Feldman R. (2003). Infant–mother and infant–father synchrony: the coregulation of positive arousal. Infant Ment. Health J. 24, 1–23. doi: 10.1002/imhj.10041

[ref29] Fivush R. Brotman M. A. Buckner J. P. Goodman S. H. (2000). Gender differences in parent–child emotion narratives. Sex Roles 42, 233–253. doi: 10.1023/A:1007091207068

[ref30] Fredrickson B. L. (2001). The role of positive emotions in positive psychology: the broaden-and-build theory of positive emotions. Am. Psychol. 56:218. doi: 10.1037/0003-066X.56.3.218, PMID: 11315248 PMC3122271

[ref31] Fredrickson B. L. Levenson R. W. (1998). Positive emotions speed recovery from the cardiovascular sequelae of negative emotions. Cogn. Emot. 12:191. doi: 10.1080/026999398379718, PMID: 21852890 PMC3156608

[ref32] Garcia F. Serra E. Garcia O. F. Martinez I. Cruise E. (2019). A third emerging stage for the current digital society? Optimal parenting styles in Spain, the United States, Germany and Brazil. Int. J. Environ. Res. Public Health 16:13. doi: 10.3390/ijerph16132333 PMC6651095 31269653

[ref33] Gong X. Wang C. (2023). Interactive effects of parental psychological control and autonomy support on emerging adults’ emotion regulation and self-esteem. Curr. Psychol. 42, 16111–16120. doi: 10.1007/s12144-021-01483-3, PMID: 41042414

[ref34] Green D. S. Goldstein A. L. Zhu J. Y. Hamza C. A. Scharfe E. Molnar D. S. (2024). Parents’ influences on well-being in emerging adulthood: the role of basic psychological needs. J. Child Fam. Stud. 33:10. doi: 10.1007/s10826-024-02912-0

[ref35] Grolnick W. S. Lerner R. E. (2023). “How parental autonomy support, structure and involvement help children flourish: considering interactions, context and diversity” in Oxford Handb. Self-Determin. Theory. ed. Ryan R. M. (Oxford: OUP), 491–508.

[ref36] Gross J. J. (2015). Emotion regulation: current status and future prospects. Psychol. Inq. 26, 1–26. doi: 10.1080/1047840X.2014.940781

[ref37] Hernandez Hernandez M. E. Taşkesen N. Van der Kaap-Deeder J. (2025). Emotion crafting and daily psychological functioning: a seven-day diary study. J. Happiness Stud. 26:30. doi: 10.1007/s10902-025-00876-6

[ref38] Hofstede G. Hofstede G. J. Minkov M. (2010). Cultures and organisations: Software of the mind. 3rd Edn. New York: McGraw-Hill.

[ref39] Hu L. Bentler P. M. (1999). Cut-off criteria for fit indexes in covariance structure analysis: conventional criteria versus new alternatives. Struct. Equ. Model. 6, 1–55. doi: 10.1080/10705519909540118

[ref40] IBM Corp (2021). IBM SPSS statistics for windows (version 28.0). Armonk, NY: IBM Corp.

[ref41] Inguglia C. Ingoglia S. Liga F. Lo Coco A. Lo Cricchio M. G. (2015). Autonomy and relatedness in adolescence and emerging adulthood: relationships with parental support and psychological distress. J. Adult Dev. 22:1. doi: 10.1007/s10804-014-9196-8

[ref42] Jaffe M. Gullone E. Hughes E. K. (2010). The roles of temperamental dispositions and perceived parenting behaviours in the use of two emotion regulation strategies in late childhood. J. Appl. Dev. Psychol. 31:1. doi: 10.1016/j.appdev.2009.07.008

[ref43] Kerr D. C. R. Capaldi D. M. (2019). “Intergenerational transmission of parenting” in Handbook of parenting: Being and becoming a parent. ed. Bornstein M. H.. 3rd ed (New York: Routledge/Taylor & Francis), 443–481.

[ref44] Kerr D. C. R. Capaldi D. M. Pears K. C. Owen L. D. (2009). A prospective three generational study of fathers’ constructive parenting: influences from family of origin, adolescent adjustment, and offspring temperament. Dev. Psychol. 45, 1257–1275. doi: 10.1037/a0015863, PMID: 19702390 PMC2742381

[ref45] Khaleque A. (2013). Perceived parental warmth, and children’s psychological adjustment, and personality dispositions: a meta-analysis. J. Child Fam. Stud. 22:2. doi: 10.1007/s10826-012-9579-z

[ref46] Klimes-Dougan B. Brand A. E. Zahn-Waxler C. Usher B. Hastings P. D. Kendziora K.. (2007). Parental emotion socialisation in adolescence: differences in sex, age and problem status. Soc. Dev. 16:2. doi: 10.1111/j.1467-9507.2007.00387.x

[ref47] Kline R. B. (2015). Principles and practice of structural equation modeling. 4th Edn. New York: Guilford Press.

[ref48] Korelitz K. E. Garber J. (2016). Congruence of parents’ and children’s perceptions of parenting: a meta-analysis. J. Youth Adolesc. 45:1973. doi: 10.1007/s10964-016-0524-0, PMID: 27380467 PMC5222679

[ref49] Krebs P. Norcross J. C. Nicholson J. M. Prochaska J. O. (2018). Stages of change and psychotherapy outcomes: a review and meta-analysis. J. Clin. Psychol. 74, 1964–1979. doi: 10.1002/jclp.22683, PMID: 30335193

[ref50] Larsen R. J. Buss D. M. Wismeijer A. Song J. Van den Berg S. Jeronimus B. F. (2025). Personality psychology: Domains of knowledge about human nature. New York: McGraw-Hill.

[ref51] Livingstone K. M. Srivastava S. (2012). Up-regulating positive emotions in everyday life: strategies, individual differences and associations with positive emotion and well-being. J. Res. Pers. 46:5. doi: 10.1016/j.jrp.2012.05.009

[ref52] Lynam D. R. Hoyle R. H. Newman J. P. (2006). The perils of partialling: cautionary tales from aggression and psychopathy. Assessment 13:3. doi: 10.1177/1073191106290562 16880283 PMC3152746

[ref9001] Ma X. Tamir M. Miyamoto Y. (2018). A socio-cultural instrumental approach to emotion regulation: culture and the regulation of positive emotions. Emotion 18:1. doi: 10.1037/emo0000315 28414476

[ref53] Majdandžić M. Möller E. L. de Vente W. Bögels S. M. van den Boom D. C. (2014). Fathers’ challenging parenting behavior prevents social anxiety development in their 4-year-old children: a longitudinal observational study. J. Abnorm. Child Psychol. 42, 301–310. doi: 10.1007/s10802-013-9774-4, PMID: 23812638

[ref54] Martins-Klein B. Alves L. A. Chiew K. S. (2020). Proactive versus reactive emotion regulation: a dual-mechanisms perspective. Emotion 20, 87–92. doi: 10.1037/emo0000664, PMID: 31961184

[ref55] McDonald R. P. (1999). Test theory: A unified treatment. Mahwah, NJ: Lawrence Erlbaum Associates.

[ref56] Mesquita B. Boiger M. De Leersnyder J. (2017). Doing emotions: the role of culture in everyday emotions. Eur. Rev. Soc. Psychol. 28:1. doi: 10.1080/10463283.2017.1329107

[ref57] Miyamoto Y. Ma X. Wilken B. (2017). Cultural variation in pro-positive versus balanced systems of emotions. Curr. Opin. Behav. Sci. 15, 27–32. doi: 10.1016/j.cobeha.2017.05.014

[ref58] Moran K. M. Turiano N. A. Gentzler A. L. (2018). Parental warmth during childhood predicts coping and well-being in adulthood. J. Fam. Psychol. 32:610. doi: 10.1037/fam0000401, PMID: 29708363 PMC6072567

[ref59] Morris A. S. Criss M. M. Silk J. S. Houltberg B. J. (2017). The impact of parenting on emotion regulation during childhood and adolescence. Child Dev. Perspect. 11:4. doi: 10.1111/cdep.12238

[ref60] Morris A. S. Ratliff E. L. Cosgrove K. T. Steinberg L. (2021). We know even more things: a decade review of parenting research. J. Res. Adolesc. 31, 870–888. doi: 10.1111/jora.12641, PMID: 34820951 PMC8630733

[ref61] Morris A. S. Silk J. S. Steinberg L. Myers S. S. Robinson L. R. (2007). The role of the family context in the development of emotion regulation. Soc. Dev. 16, 361–388. doi: 10.1111/j.1467-9507.2007.00389.x, PMID: 19756175 PMC2743505

[ref62] Nelson L. J. (2022). “Parenting during emerging adulthood” in The Cambridge handbook of parenting: Interdisciplinary research and application. eds. Morris A. S. Mendez Smith J. (Cambridge: Cambridge University Press), 259–278.

[ref63] Neppl T. K. Conger R. D. Scaramella L. V. Ontai L. L. (2009). Intergenerational continuity in parenting behavior: mediating pathways and child effects. Dev. Psychol. 45, 1241–1256. doi: 10.1037/a0014850, PMID: 19702389 PMC2748920

[ref64] Nunnally J. C. Bernstein I. H. (1994). Psychometric theory. 3rd Edn. New York, NY: McGraw-Hill.

[ref65] Pepping C. A. Duvenage M. (2016). The origins of individual differences in dispositional mindfulness. Pers. Individ. Differ. 93, 130–136. doi: 10.1016/j.paid.2015.05.027

[ref66] Posit Team (2023). RStudio: Integrated development environment for R. Boston, MA: Posit Software PBC.

[ref67] Pressman S. D. Cohen S. (2005). Does positive affect influence health? Psychol. Bull. 131, –971. doi: 10.1037/0033-2909.131.6.925, PMID: 16351329

[ref68] Quach A. S. Epstein N. B. Riley P. J. Falconier M. K. Fang X. (2015). Effects of parental warmth and academic pressure on anxiety and depression symptoms in Chinese adolescents. J. Child Fam. Stud. 24:1. doi: 10.1007/s10826-013-9818-y

[ref69] Quoidbach J. Mikolajczak M. Gross J. J. (2015). Positive interventions: an emotion regulation perspective. Psychol. Bull. 141, 655–693. doi: 10.1037/a0038648, PMID: 25621978

[ref70] Reitsema A. M. Jeronimus B. F. van Dijk M. de Jonge P. (2022). Emotion dynamics in children and adolescents: a meta-analytic and descriptive review. Emotion 22:2. doi: 10.1037/emo0000970 34843305

[ref71] Richard-Sephton P. B. Crisp D. A. Burns R. A. (2024). The emotion regulation strategies of flourishing adults. Curr. Psychol. 43:12816. doi: 10.1007/s12144-023-05332-3, PMID: 41042414

[ref72] Robbins R. J. (1994). An assessment of perceptions of parental autonomy support and control: child and parent correlates. Rochester, NY: Univ. Rochester.

[ref73] Ryan R. M. Deci E. L. Vansteenkiste M. (2016). “Autonomy and autonomy disturbances in self-development and psychopathology: research on motivation, attachment and clinical process” in Dev. Psychopathol.: Theory method. ed. Cicchetti D.. 3rd ed (Hoboken, NJ: Wiley), 385–438.

[ref74] Schofield T. J. Martin M. Conger R. D. Neppl T. K. Donnellan M. B. Conger K. J. (2011). Intergenerational transmission of adaptive functioning: a test of the interactionist model of SES and human development. Child Dev. 82, 33–47 doi: 10.1111/j.1467-8624.2010.01539.x 21291427 PMC3058312

[ref75] Shulman S. Connolly J. (2013). The challenge of romantic relationships in emerging adulthood: reconceptualization of the field. Emerg. Adulthood 1:1. doi: 10.1177/2167696812467330

[ref76] Sigelman C. K. Rider E. A. (2014). Life-span human development. 8th Edn. Boston, MA: Cengage Learning.

[ref77] Slemp G. R. Field J. G. Ryan R. M. Forner V. W. Van den Broeck A. Lewis K. J. (2024). Interpersonal supports for basic psychological needs and their relations with motivation, well-being and performance: a meta-analysis. J. Pers. Soc. Psychol. 127:1012. doi: 10.1037/pspi0000459, PMID: 38635183

[ref78] Smith B. W. Dalen J. Wiggins K. Tooley E. Christopher P. Bernard J. (2008). The brief resilience scale: assessing the ability to bounce back. Int. J. Behav. Med. 15, 194–200. doi: 10.1080/10705500802222972, PMID: 18696313

[ref79] Snyder J. J. (2016). “Coercive family processes and the development of child social behavior” in The Oxford handbook of coercive relationship dynamics. eds. Dishion T. J. Snyder J. J. (New York: Oxford Univ. Press), 101–113.

[ref80] Soenens B. Deci E. L. Vansteenkiste M. (2017). “How parents contribute to children’s psychological health: the critical role of psychological need support” in Development of self-determination through the life-course. eds. Wehmeyer M. L. Shogren K. A. Little T. D. Lopez S. J. (Cham: Springer), 171–187.

[ref81] Song H. Chan J. S. Ryan C. (2024). Differences and similarities in the use of nine emotion regulation strategies in Western and east-Asian cultures: systematic review and meta-analysis. J. Cross-Cult. Psychol. 55:8. doi: 10.1177/00220221241285006

[ref82] Tabachnick B. G. Fidell L. S. (2013). Using multivariate statistics. 6th Edn. Boston, MA: Pearson.

[ref83] Tan P. Wang R. Long T. Wang Y. Ma C. Ma Y. (2024). Associations between parental autonomy support and depressive symptoms among Chinese college students: the chain-mediating effects of mindfulness and self-esteem. Front. Psychol. 15:1301662. doi: 10.3389/fpsyg.2024.1301662, PMID: 38778882 PMC11110894

[ref84] Tani F. Pascuzzi D. Raffagnino R. (2018). The relationship between perceived parenting style and emotion regulation abilities in adulthood. J. Adult Dev. 25:1. doi: 10.1007/s10804-017-9269-6

[ref85] Turner J. Roberts R. M. Proeve M. Chen J. (2023). Relationship between PERMA and children’s wellbeing, resilience and mental health: a scoping review. Int. J. Wellbeing 13, 20–44. doi: 10.5502/ijw.v13i2.2515

[ref86] Ullman J. B. (2001). “Structural equation modelling” in Using multivariate statistics. eds. Tabachnick B. G. Fidell L. S.. 4th ed (Boston, MA: Allyn & Bacon), 653–771.

[ref87] Van der Kaap-Deeder J. Vansteenkiste M. Soenens B. Mabbe E. (2017). Children’s daily well-being: the role of mothers’, teachers’ and siblings’ autonomy support and psychological control. Dev. Psychol. 53:2. doi: 10.1037/dev0000218 27736100

[ref88] Van der Kaap-Deeder J. Wichstrøm L. Mouratidis A. Matos L. Steinsbekk S. (2023). Emotion crafting: individuals as agents of their positive emotional experiences. Motiv. Emot. 47, 870–886. doi: 10.1007/s11031-023-10035-0

[ref89] Van Lissa C. J. Keizer R. Van Lier P. A. C. Meeus W. H. J. Branje S. (2019). The role of fathers’ versus mothers’ parenting in emotion-regulation development from mid- to late adolescence: disentangling between-family differences from within-family effects. Dev. Psychol. 55:2. doi: 10.1037/dev0000612 30570297

[ref91] Yavuz H. M. Colasante T. Malti T. (2022). Parental warmth predicts more child pro-social behaviour in children with better emotion regulation. Br. J. Dev. Psychol. 40:539. doi: 10.1111/bjdp.12425, PMID: 35751141

[ref92] Yoon M. Lai M. H. C. (2018). Testing factorial invariance with unbalanced samples. Struct. Equ. Model. 25:2. doi: 10.1080/10705511.2017.1387859

[ref93] Yu J. Putnick D. L. Hendricks C. Bornstein M. H. (2019). Long-term effects of parenting and adolescent self-competence for the development of optimism and neuroticism. J. Youth Adolesc. 48:1544. doi: 10.1007/s10964-018-0980-9, PMID: 31111366 PMC6643290

[ref94] Zhou Q. Eisenberg N. Losoya S. H. Fabes R. A. Reiser M. Guthrie I. K.. (2002). The relations of parental warmth and positive expressiveness to children’s empathy-related responding and social functioning: a longitudinal study. Child Dev. 73:893. doi: 10.1111/1467-8624.00446, PMID: 12038559

[ref95] Zimmermann P. Iwanski A. (2014). Emotion regulation from early adolescence to emerging adulthood and middle adulthood: age differences, gender differences, and emotion-specific developmental variations. Int. J. Behav. Dev. 38:2. doi: 10.1177/0165025413515405

